# Ultrafine particles cause cytoskeletal dysfunctions in macrophages: role of intracellular calcium

**DOI:** 10.1186/1743-8977-2-7

**Published:** 2005-10-04

**Authors:** Winfried Möller, David M Brown, Wolfgang G Kreyling, Vicki Stone

**Affiliations:** 1 GSF National Research Center for Environment and Health, Clinical research group 'Inflammatory Lung Diseases', Robert Koch Allee 29, D-82131 Munich-Gauting, Germany; 2 GSF National Research Center for Environment and Health, Institute for Inhalation Biology, and Focus Network Aerosols and Health, Ingolstädter Landstr. 1, D-85746 Neuherberg/München, Germany; 3 Napier University, School of Life Sciences, Edinburgh EH10 5DT, UK

**Keywords:** Ultrafine particles, cytoskeleton, stiffness, viscoelasticity, phagosome transport, relaxation, intracellular calcium

## Abstract

**Background:**

Particulate air pollution is reported to cause adverse health effects in susceptible individuals. Since most of these particles are derived form combustion processes, the primary composition product is carbon with a very small diameter (ultrafine, less than 100 nm in diameter). Besides the induction of reactive oxygen species and inflammation, ultrafine particles (UFP) can cause intracellular calcium transients and suppression of defense mechanisms of alveolar macrophages, such as impaired migration or phagocytosis.

**Methods:**

In this study the role of intracellular calcium transients caused by UFP was studied on cytoskeleton related functions in J774A.1 macrophages. Different types of fine and ultrafine carbon black particles (CB and ufCB, respectively), such as elemental carbon (EC90), commercial carbon (Printex 90), diesel particulate matter (DEP) and urban dust (UD), were investigated. Phagosome transport mechanisms and mechanical cytoskeletal integrity were studied by cytomagnetometry and cell viability was studied by fluorescence microscopy. Macrophages were exposed in vitro with 100 and 320 *μ*g UFP/ml/million cells for 4 hours in serum free medium. Calcium antagonists Verapamil, BAPTA-AM and W-7 were used to block calcium channels in the membrane, to chelate intracellular calcium or to inhibit the calmodulin signaling pathways, respectively.

**Results:**

Impaired phagosome transport and increased cytoskeletal stiffness occurred at EC90 and P90 concentrations of 100 *μ*g/ml/million cells and above, but not with DEP or UD. Verapamil and W-7, but not BAPTA-AM inhibited the cytoskeletal dysfunctions caused by EC90 or P90. Additionally the presence of 5% serum or 1% bovine serum albumin (BSA) suppressed the cytoskeletal dysfunctions. Cell viability showed similar results, where co-culture of ufCB together with Verapamil, W-7, FCS or BSA produced less cell dead compared to the particles only.

## Background

Epidemiological studies suggest increased health risks (decreased lung function, increased morbidity and mortality) after exposure to environmental particles [1,2]. Recent epidemiological studies show, that not only the mass of inhaled urban particles (i.e. PM10 or PM2.5 ≡ mass of all particles being smaller than 10 *μ*m or 2.5 *μ*m, respectively) is associated with increased morbidity and mortality, but also the number of particles with even higher significance [3,4]. Because of the smaller mass of ultrafine (diameter < 100 nm) compared to fine particles, they contribute little to the total mass, but are important in the overall number of particles. Most urban particles result from combustion processes; therefore the dominant fraction contains ultrafine carbonaceous particles. These particles are less soluble and therefore can reside in the lung for longer times. Larger particles and bacteria are phagocytized by alveolar and airway macrophages and dendritic cells within hours and further digested, keeping the lung surface in a sterile condition (under physiological conditions). UFP are not only phagocytosed by alveolar macrophages, but can enter epithelial cells, can penetrate into the circulation, being further redistributed to other organs of the body [5]. Therefore, UFP can reach sites in the body far away from the site of entrance, where they can cause inflammatory reactions, in contrast to larger particles [6,7]. With decreasing particle size the particle surface increases in relation to the particle volume or particle mass. Particles forming aggregates being composed of subunits of much smaller size can further enhance their surface area in relation to the mass (specific surface area). Because of their high specific surface area, UFP can catalyze chemical reactions, which may induce chronic inflammation. Animal studies and in vitro cell studies have shown that high concentrations of ultrafine particles can induce inflammatory processes and increased calcium transients [8-13], can induce the production of reactive oxygen species (ROS, like H_2_O_2_, O_2_^-^) [14-16], can induce oxidation of cytoskeletal proteins [17,18] and cytoskeletal dysfunctions [19,20], or can act as vehicles to transport toxic gasses and substances to the lung periphery [21].

Phagocytic cells in the lung, such as macrophages, polymorphonuclear leukocytes and dendritic cells play a key-role in the defense-reaction. They ingest foreign materials, digest bacteria and viruses, and present antigens in order to trigger specific (immunological) defense mechanisms [22-24]. Macrophages reside in the alveoli and chemotactic gradients direct the macrophages within minutes to the site of particle deposition. During phagocytosis the particles are incorporated into a membranous vesicle and ingested into intracellular phagosomes, which fuse with lysosomes [25]. Phagolysosomes are acidic (pH ≈ 5) and contain reactive oxygen species (H_2_O_2_) [26,27]. They are the site where most bacteria and fungi are digested. Non-digestible particles are retained in the lung for longer periods of time, for example iron oxide tracer particles have a clearance half-time of 120 days in healthy non-smoking subjects [28]. UFP are not only engulfed by phagocytosis and therefore must not necessarily reside in phagolysosomes, which may imply much longer residence times in the lung. The cytoskeleton of the AM is crucially involved in the phagocytic defense reactions, including locomotion and cell migration, phagocytosis, intracellular transport, phagosome-lysosome fusion and signal transduction [29-31]. The cytoskeleton consists of three different filamentous structures. Microfilaments (actin) are involved in dynamic processes of the cell, like crawling and phagocytosis. Microtubuli are involved in cell shape and intracellular vesicle transport. Intermediate filaments contribute to the static part of the cytoskeleton. Every filamentous structure has its own family of motor proteins being responsible for the transport of molecules and vesicles [32]. Those transport processes require energy in the form of ATP [33,34]. The cytoskeleton is sensitive to ROS and oxidative stress, due to the presence of thiol groups located on the actin microfilaments which are sensitive to oxidation, leading to cross linking and reduced motility [18,35], and the cytoskeletal dynamics is regulated by cytoplasmic calcium.

We have developed protocols to study cytoskeleton associated functions in macrophages in vivo and in vitro, such as phagosome transport, mechanical integrity (viscoelastic properties, stiffness) and phagocytosis, using ferromagnetic microparticles [36-38]. In *in vitro *studies the magnetic microparticles (1.8 *μ*m diameter) are incubated for 24 hours together with cultivated macrophages, after which more than 95% are ingested. The particles are then magnetized and aligned in a short magnetic field pulse, and can be detected by a magnetic field sensor. Intracellular phagosome transport causes stochastic disorientations of the particles, which results in a decay of the remanent magnetic field of the cell probe (relaxation). In addition mechanical viscoelastic properties of the cytoskeleton can be investigated by twisting the micromagnets in a weak magnetic field. This method is called 'Magnetic Twisting Cytometry' (MTC) [39,40] and has been used to investigate the role of the different cytoskeletal structures in macrophage function after co-incubation with cytoskeletal drugs (Cytochalasin D, Nocodazole [37]). Additionally it has been shown that MTC can monitor cytotoxicity of GaAs-particles *in vivo *in lung macrophages of animals [41,42] and in vitro caused by ultrafine particles [20].

In this study, we have investigated the role of intracellular calcium transients produced by environmentally relevant fine and ultrafine model particles in the induction of adverse reactions on the cytoskeleton of macrophages, using the MTC technique. Cultivated macrophages from the cell line J774A.1 were incubated with increasing amounts of particles. Intracellular phagosome transport (relaxation), stiffness of the cytoskeleton and cell viability was recorded. Particles on the basis of carbon black (CB) having different chemical compositions and impurities, and a wide range of diameters (12 nm - 1.5 *μ*m) and specific surface areas (≈ 1 m^2^/g - 600 m^2^/g) were tested.

## Materials and methods

### Target cells

J774A.1 macrophages originate from a BALB/c/NIH mouse [43] and were obtained from the German Collection of Animal Cell Cultures (Tumorbank, DKFZ Heidelberg, Germany). Cells were grown in RPMI 1640 Medium (Sigma, Taufkirchen, Germany), supplemented with 5% fetal calf serum (FCS), 100 U/ml penicillin, 100 *μ*g/ml streptomycin, 2.5 *μ*g/ml amphotericin and 0.3 g/L-glutamine in NaHCO_3_. The cell culture grows with a doubling time of 2 days and was sub-cultured every 4 days. In cytotoxicity studies cells were incubated with particles and with Calcium agonists in serum-free medium. In addition cells were incubated with 5% FCS to test the possible cytotoxic effect of lack of serum in the medium.

### Magnetic particle binding assay

A number of 0.2 million macrophages were incubated with 10 *μ*g of 1.8 *μ*m spherical ferromagnetic microparticles in glass vials (12 mm outer diameter) for 24 hours prior to adding calcium antagonists or ultrafine particles. This ensured the adherence of the macrophages and more than 95% phagocytosis of the magnetic beads. Before the addition of UFP or calcium antagonists, non-adherent cells and free particles were removed by washing with medium. Ferromagnetic 1.8 *μ*m magnetite microparticles (beads) were prepared with narrow size variation (geometric standard deviation < 1.1) and spherical shape [44], which is important for data analysis using mathematical models to estimate the viscous and elastic properties of the cytoskeleton. The particles were not further coated in order to avoid the activation of specific cell surface receptors. It is reported that the non-specific Scavenger-receptor mediates phagocytosis of the magnetite particles [45].

### Ultrafine test-particles and calcium modulating drugs

Table 1 gives an overview of the test particles used in the study, together with their physical properties. Because most urban particles result from combustion processes, the dominant fraction contains fine and ultrafine carbonaceous particles. Ultrafine carbon (EC90) particles were produced by an electrical spark generator [46] under standardized conditions with low impurities (for example transition metals, polycyclic hydrocarbons; about 5% organic carbon). The mobility diameter in air of these particles was 90 nm and the high specific surface area of 600 m^2^/g indicated that aggregates had much smaller subunits. Alternatively, commercially available ultrafine carbon particles were used (Printex, P90, Degussa, Frankfurt, Germany), which have low organic impurities of (≈ 1%), but some impurities of transition metals (iron etc.). Diesel particulate matter (Standard Reference Material 1650, DEP) and urban dust (Standard Reference Material 1649a, UD) were used to simulate environmental particle exposure [47,48]. The certificate of analysis of UD does not give an estimation of the specific surface area; therefore a value from literature was obtained for a comparable urban dust (PM2.5) probe [49]. The particles were suspended within glass tubes in deionised water by ultrasonication and then further diluted in medium without serum. Microscopic investigation showed that a large fraction of the carbonaceous particles appeared as aggregates in the cell probes.

**Table 1:** Fine and ultrafine test particles used in the study together with their physical properties, such as count median diameter and specific surface area. Elemental carbon particles (EC90) were prepared in own laboratory (GSF-IHB, [46]).

**Material type**	**Diameter**	**Specific surface area**	**Source**
Printex90 (carbon, P90)	12 nm	300 m^2^/g	Degussa
Elemental carbon (EC90)	90 nm^+^	600 m^2^/g	GSF-IHB
Diesel exhaust particles (DEP)	120 nm	108 m^2^/g	NIST*
Urban dust (UD)	1.5 *μ*m	≈ 1 m^2^/g^++^	NIST*

^+ ^size of the airborne agglomerates; primary particles are expected to be < 10 nm [46].

* National Institute of Standards and Technology (NIST), Washington, USA [47, 48].

^++^estimated from other PM2.5 ([49])

Particles were incubated at concentrations of 100 and 320 *μ*g/ml/million cells for 4 h in serum-free medium, or in combination with calcium antagonists, such as Verapamil (100 *μ*M, Ca^2+ ^channel blocker), BAPTA-AM (20 *μ*M, Ca^2+ ^chelator), or W-7 (N-(6-aminohexyl)-5-chloro-1-naphthalene-sulfonamide hydrochloride, 25 *μ*M, calmodulin inhibitor). The concentration of the Ca-antagonists was obtained from previous studies and tested for non-toxic responses in own preliminary studies. The antioxidant Nacystelin (NAL, 200 *μ*M) was also included. Nacystelin (NAL), a thiol antioxidant compound that is a lysinated derivative of N-acetyl cysteine (NAC), possesses potent mucolytics capacities and has been shown to inhibit reactive oxygen species effects [50]. It has the advantage of having a neutral pH compared with NAC, which is acidic, and thus can be administered to the airways without the local airway irritation that occurs with NAC. Additionally the role of serum (FCS, 5%) or bovine serum albumin (BSA, 1%) in the medium was investigated. In some experiments long-term incubation (24 hours) was carried out to compare with previous data.

### Cell viability

Cell viability was tested by the propidium iodide (PI) exclusion test. Necrotic cells allow the penetration of PI into the cell and the nucleus, where the fluorescent dye can be visualized. After 10 min PI incubation with adherent cells, the probes were analyzed by fluorescence microscopy, using an inverted microscope (Axiovert 25, Zeiss GmbH, Jena, Germany) together with a mercury-arc lamp and a filter system for the detection of propidium iodide (PI) dye and a digital camera system (CoolSnap-pro, Mediacybernetics Inc., Silver Spring, MD, USA). Digital images were recorded and processed using Image Pro software package (Mediacybernetics Inc., Silver Spring, MD, USA). For every probe a transmission image was recorded as well as a fluorescence image. The transmission image was used to count the total number of cells in a region of interest (ROI), defined by a grid overlay and covering about 80–100% of the image. Each ROI covered at least 100 cells. In the second fluorescence image the number of PI positive cells was analyzed in the same ROI, allowing the fraction of dead cells (PI+) to be assessed.

### Magnetic twisting cytometry

**Figure 1:**
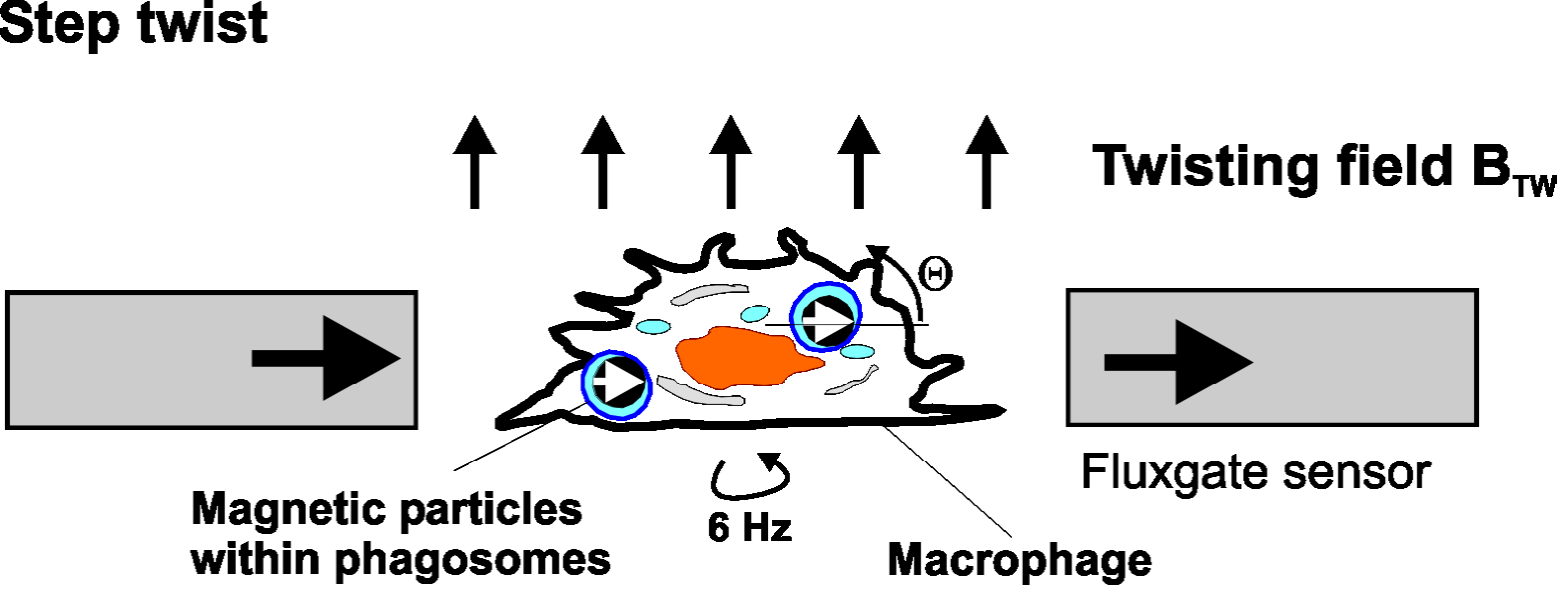
Magnetic twisting device to measure relaxation and twisting (10 sec) of aligned ferromagnetic microparticles ingested by macrophages and detection by an array of magnetic fluxgate sensors (Förster devices, Förster GmbH, Reutlingen, Germany).

For in vitro measurement of relaxation and viscoelastic cell properties a magnetic twisting cytometry device (MTC) was used [40]. A glass vial (12 mm diameter) containing adherent macrophages with ingested magnetic particles is positioned in a second gradiometer array of flux-gate sensors (Förster GmbH, Reutlingen, Germany; Figure 1). The particles in the cells were aligned parallel to the direction of the sensors by a 200 mT, 10 *μ*s magnetic field pulse. The probe was rotated at 6 Hz and the signal of the fluxgate array was amplified and phase sensitive detected which significantly reduced system noise and improved the sensitivity. 10 *μ*g of ferromagnetic particles induce a remanent magnetic field (RMF) of ≈ 1 nT in the sensor array. Particle twisting was performed in a magnetic twisting field (1 – 2, 5 mT) perpendicular to the direction of detection (parallel to the axis of rotation).

### Stochastic phagosome motion (relaxation)

**Figure 2:**
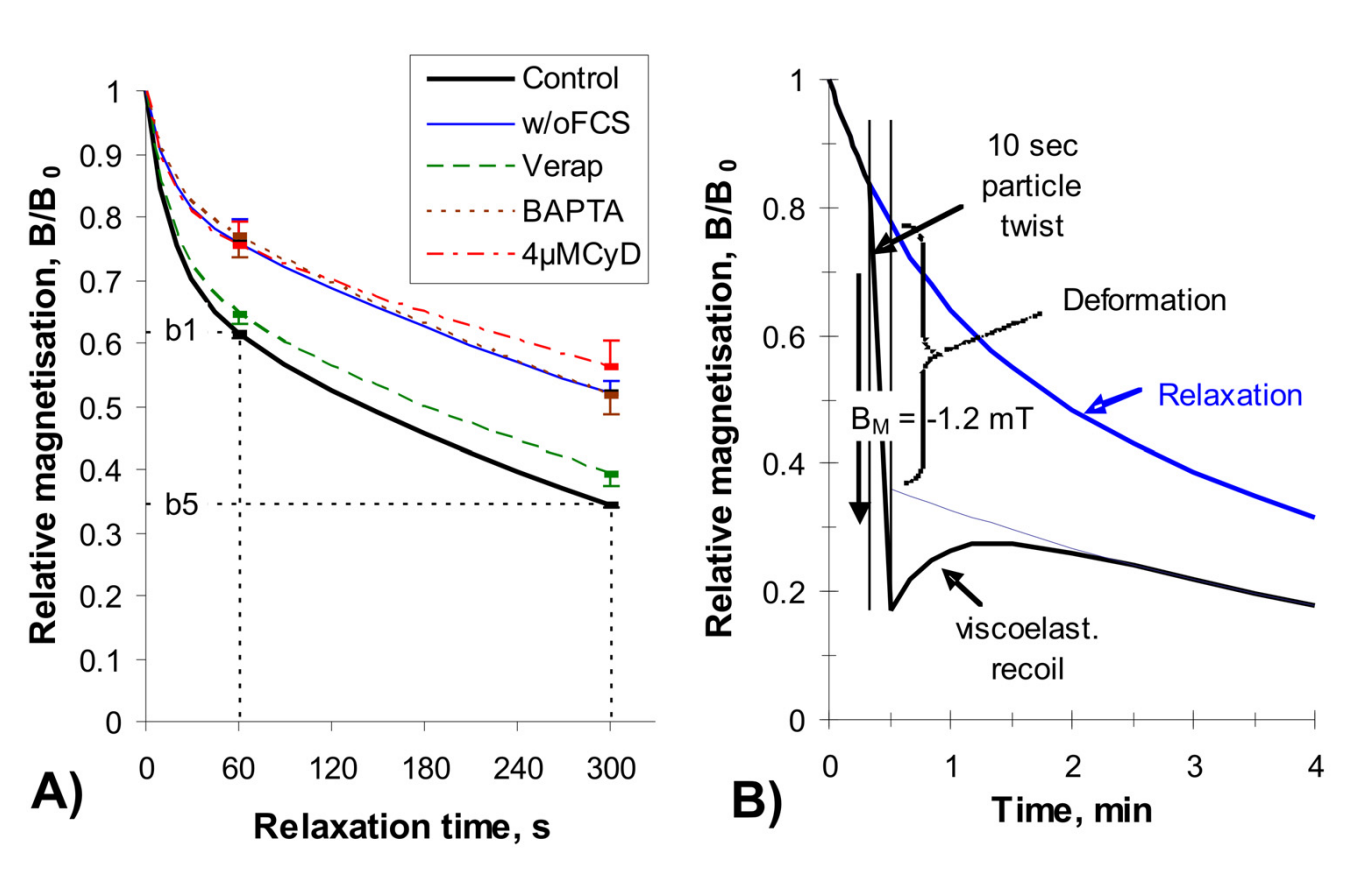
Measurement of stochastic intracellular phagosome transport (relaxation, A) and cytoskeletal stiffness (B) by Magnetic Twisting Cytometry (MTC). Control and cytochalasin D (CyD) probes do not contain UFP. Other probes show co-incubation with 100 *μ*g/ml P90 without serum (w/o FCS) and with 100 *μ*M Verapamil or 20 *μ*M BAPTA-AM for 4 hours.

The motion of vesicles and phagosomes happens continuously within living cells and is part of the intracellular transport system. Relaxation describes the decay of the magnetic field after particle alignment in a magnetic pulse field and originates from the randomization of the magnetic particles. We assume that the phagosomes are coupled to the cytoskeletal filaments by motor proteins and that the hydrolization of ATP provides the energy to move the phagosomes and to induce rotational random kicks to the phagosome [33,39,51,52]. A hydrodynamic relaxation model was developed under the assumption that the intracellular randomization energy E_r _behaves like thermal energy kT, implying a rotational Brownian motion process together with an exponential decay in a Newtonian viscosity. The hydrodynamic relaxation model was fitted to the experimental data by a non-linear regression algorithm. Additionally two robust relaxation parameters were analyzed, being independent of any model (Figure 2A). This is the normalized RMF after 1 minute, b1 = B(1 min)/B_0_, which characterizes the initial fast phase of decay, and that after 5 minutes, b5 = B(5 min)/B_0_, which is characteristic for the decay in the following slow phase. Figure 2A illustrates the decay of aligned particles and the impairment by UFP and by Cytochalasin D, a microfilament disruption agent.

### Magnetic phagosome twisting

Application of a weak magnetic field B_TW _induces twisting of a magnetic dipole particle and allows investigating cytoplasmic rheology and mechanical integrity [36,53]. Particles being suspended in a viscosity *η *rotate in an external twisting force according to Newton's law:



where dθ/dt is the shear rate and *σ *is the applied shear stress (, *M *= remanent magnetization of particles, κ = rotational shape factor). In case of elasticity the applied stress is proportional to the elastic deformation (strain θ) and we get with the elasticity modulus ν Hook's law:

*σ *= νθ (2)

Strain was estimated from the measurement of the cell field B(t) according to θ(*t*) = arccos *B*(*t*)/*B*_0_. First the particles were magnetized and aligned parallel to the field sensors (Figure 2B). After 20 sec relaxation the twisting field was applied for 10 sec duration and viscoelastic recoil was recorded for another 3 minutes. Viscoelastic recoil does not force the dipoles back to the undisturbed relaxation curve, indication of viscous shear, which can reflect a permanent deformation (break of filamentous interactions) of the cytoskeletal structure. Figure 2B shows the change of particle alignment during magnetic field twisting together with the elastic recoil in comparison to a relaxation curve recorded without any external forces to the magnetic beads. Permanent deformation is characterized by the difference in complete elastic recoil and undisturbed relaxation, as illustrated in Figure 2B. Cell stiffness was estimated as the ratio between mean stress and strain after a constant twisting duration of 10 sec. This analysis of particle twisting does not view specific viscous or elastic properties. Therefore this parameter provides an integral description of the cytoskeletal mechanical properties.

### Data analysis

**Figure 3:**
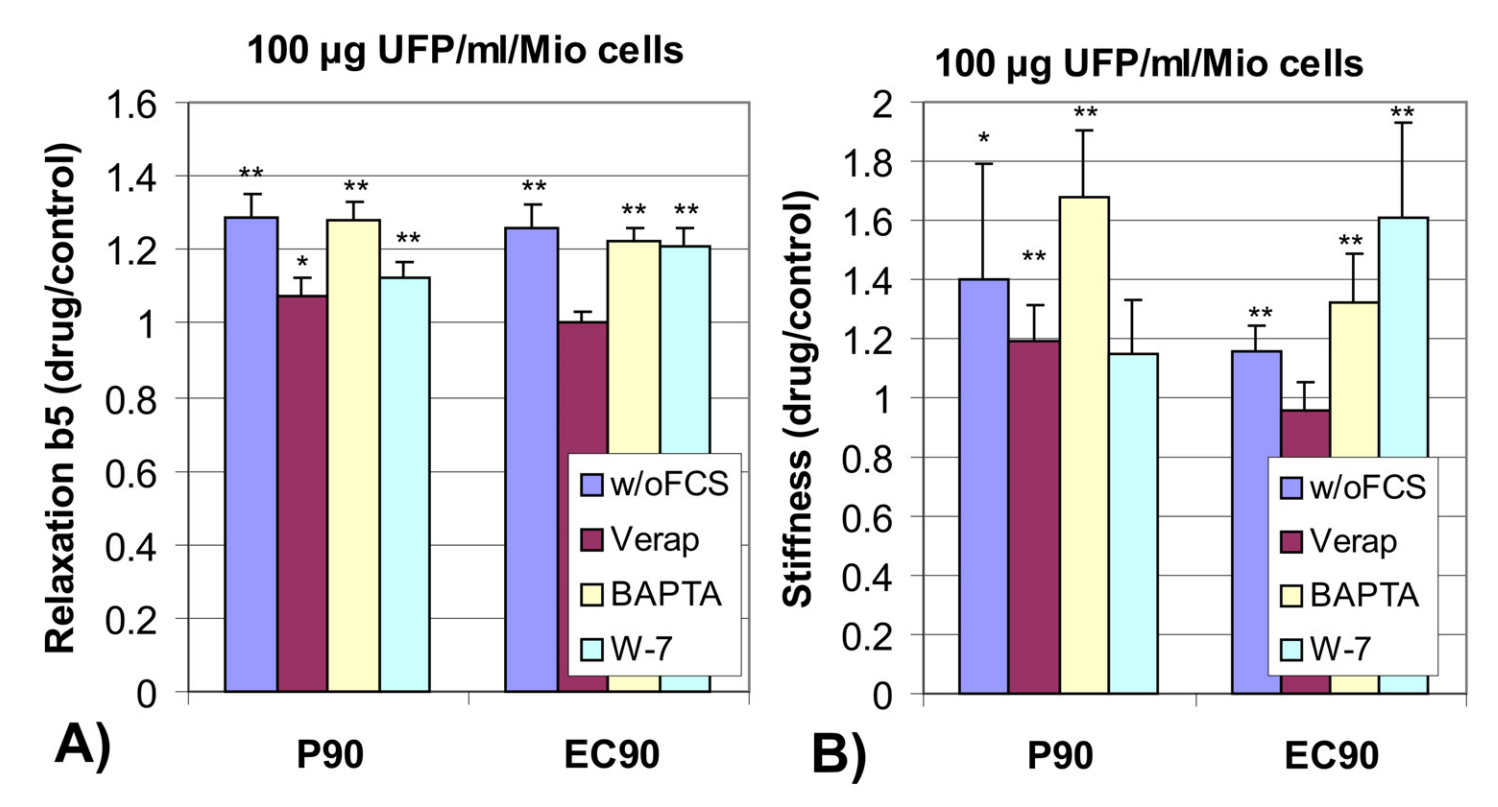
Relaxation of ingested magnetic particles (A, relative decay after 5 min, b5, normalized to control probes without particles) and mechanical integrity (B, stiffness, mean between low and high stress) of J774A.1 macrophages after 4 h incubation with different types of 100 *μ*g ultrafine particles/ml/million cells under different incubation conditions, such as without serum (w/o FCS), 100 *μ*M Verapamil, 20 *μ*M BAPTA-AM or 25 *μ*M W-7 (normalized values +/- SD; N = 5; **: p < 0.01, *: p < 0.05).

**Figure 4:**
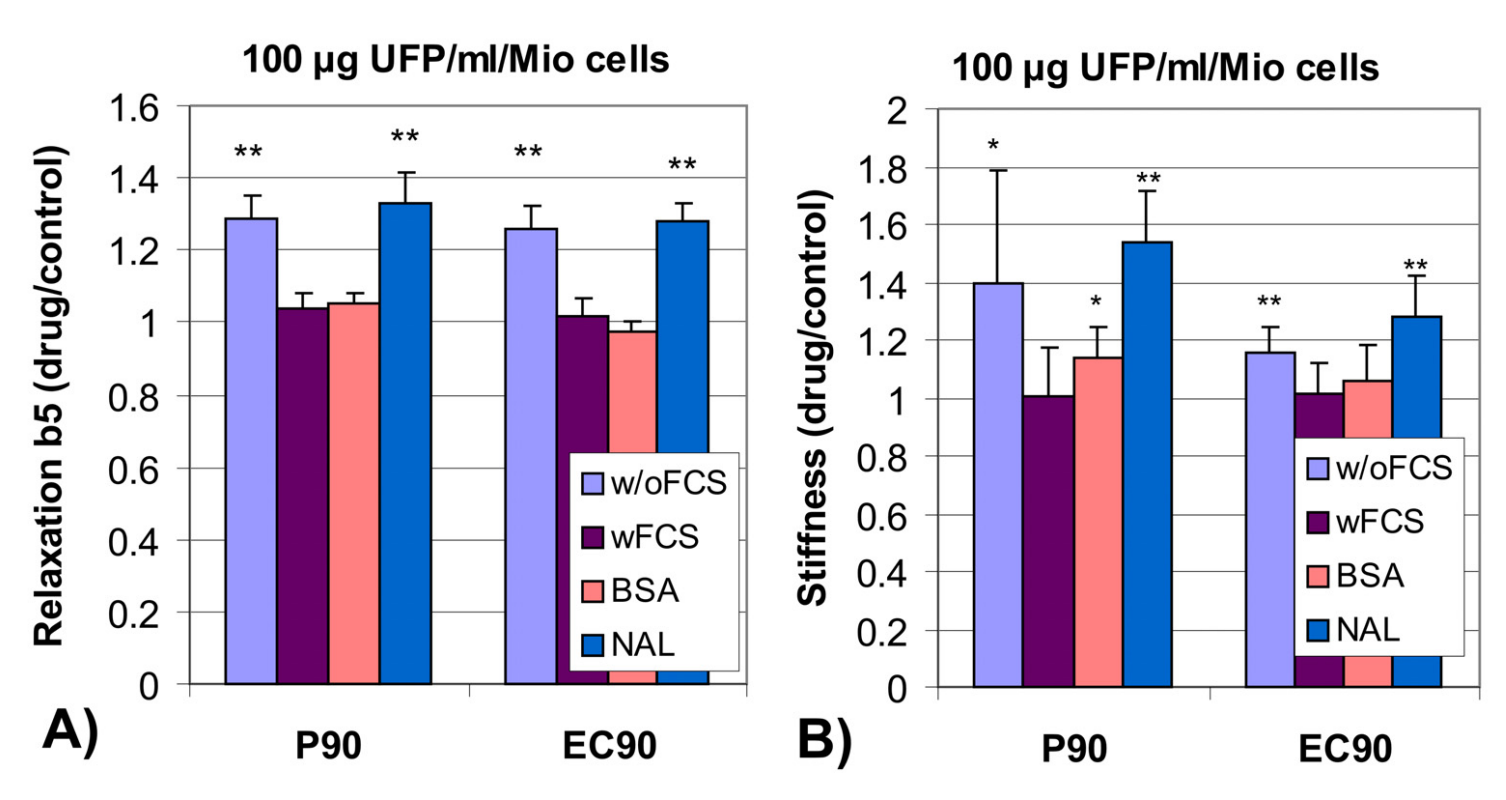
Relaxation of ingested magnetic particles (A, relative decay after 5 min, b5, normalized to control probes w/o particles) and mechanical integrity (B, stiffness, mean between low and high stress) of J774A.1 macrophages after 4 h incubation with different types of 100 *μ*g ultrafine particles/ml/million cells under different incubation conditions, such as without (w/o) and with 5% serum (wFCS), 1% bovine serum albumin (BSA) or 200 *μ*M Nacystelin (NAL); normalized values +/- SD; N = 5; **: p < 0.01, *: p < 0.05.

The data presented in Figures 3 and 4 under the influence of particles or drugs are normalized to results of control probes without particle co-incubation. An inhibited relaxation (slower decay, higher b5) results then in a normalized value larger than one. An accelerated relaxation displays as a normalized value smaller than one. Every set of UFP/calcium antagonist measurements was performed on at least 5 separate probes together with a separate set of control measurements. All parameters estimated under the set of antagonists were normalized to the set of control measurements. A significant deviation of the normalized parameters from unity denotes an influence of the UFP/antagonist. Using a 2-sided Student's t-Test, deviations from unity were analyzed for their level of statistical significance. An influence by the appropriate antagonist was accepted when the level of significance was p < 0.05. Pearson's correlation analysis was performed using WINSTAT, Version 2001.1, Fitch Software, Cambridge, USA.

## Results

### Influence of UFP on phagosome motion and on magnetic particle twisting

The results of stochastic intracellular phagosome transport and of cytoskeletal mechanical integrity after 4 hours of incubation of J774A.1 macrophages with 100 *μ*g/ml fine or ultrafine particles are shown in Figure 3 (summarized in Table 2 and Table 3). Compared to cells without particles (control), P90 and EC90 cause a retardation of relaxation both, for concentrations of 100 *μ*g/ml and of 320 *μ*g/ml (data not shown). This retardation is not seen for DEP or UD particles. The retardation of relaxation can be inhibited in part by the co-incubation with Verapamil, but not with BAPTA-AM. Co-incubation of W-7 with 100 *μ*g/ml P90 in part inhibits the retardation of relaxation, while it does not with 100 *μ*g/ml EC90 particles. UD significantly retarded relaxation only at the high concentration of 320 *μ*g/ml; the retardation could be inhibited by the calcium antagonists. The measurements of cytoskeletal stiffness (Figure 3B) reflect the same results as were found on relaxation measurements. P90 and EC90 cause an increase of cytoskeletal stiffness, when no serum is present. Verapamil lower the stiffening caused by P90 or by EC90. W-7 inhibited the stiffening only for P90 co-incubation, but not with EC90. BAPTA-AM did not influence the particle induced stiffening for both particle types. The higher particle concentration of 320 *μ*g UFP/ml reflects a comparable response. The urban particles (DEP or UD) do not modulate cytoskeletal stiffness, neither alone or in combination with calcium antagonists (data not shown). In summary the data show cytoskeletal dysfunctions caused by P90 and EC90, but not by DEP and by UD. Cytoskeletal dysfunctions can be inhibited in part by the Ca^2+ ^channel blocker Verapamil and by the calmodulin inhibitor W-7, but not by the Ca^2+ ^chelator BAPTA-AM.

**Table 2:** Summary of effects of 100 *μ*g particles/ml/million cells on phagosome transport (relaxation) together with effect of co-incubation with different Ca-modulating drugs (100 *μ*M Verapamil, 20 *μ*M BAPTA-AM or 25 *μ*M W-7) as well as medium with serum (wFCS), with bovine serum albumin (BSA), or with 200 *μ*M Nacystelin (NAL); inhib. – inhibition, n.e. – no effect, accel. – acceleration; N = 5; **: p < 0.01, *: p < 0.05.

Drug	**P90**	**EC90**	**DEP**	**UD**
w/oFCS	inhib.**	inhib.**	accel.*	n.e.
Verap	inhib.*	n.e.	n.e.	n.e.
BAPTA	inhib.**	inhib.**	n.e.	n.e.
W-7	inhib.**	inhib.**	n.e.	n.e.
wFCS	n.e.	n.e.	n.e.	n.e.
BSA	n.e.	n.e.	n.e.	n.e.
NAL	inhib.**	inhib.**	n.e.	n.e.

**Table 3:** Summary of effects of 100 *μ*g particles/ml/million cells on cytoskeletal stiffness together with effect of co-incubation with different Ca-modulating drugs (100 *μ*M Verapamil, 20 *μ*M BAPTA-AM or 25 *μ*M W-7) as well as medium with serum (wFCS), with bovine serum albumin (BSA), or with 200 *μ*M Nacystelin (NAL); stiff. – stiffening, n.e. – no effect; N = 5; **: p < 0.01, *: p < 0.05.

Drug	**P90**	**EC90**	**DEP**	**UD**
w/oFCS	stiff.*	stiff.**	n.e.	n.e.
Verap	stiff.*	n.e.	n.e.	n.e.
BAPTA	stiff.**	stiff.**	n.e.	n.e.
W-7	n.e.	stiff.**	n.e.	n.e.
wFCS	n.e.	n.e.	n.e.	n.e.
BSA	n.e.	n.e.	n.e.	n.e.
NAL	stiff.**	stiff.**	n.e.	n.e.

### Influence of serum, BSA and Nacystelin on phagosome motion and on magnetic particle twisting

Figure 4 shows the results of serum and of BSA on phagosome transport and on cytoskeletal stiffness after co-culture with P90 and EC90 (summary in Table 2 and Table 3). Both, 5% FCS and 1% BSA are able to inhibit the particle induced retardation of relaxation (Figure 4A) and the increase in cell stiffness (Figure 4B). The antioxidant Nacystelin does not inhibit the particle induced cytoskeletal dysfunctions. Serum, BSA and Nacystelin do not influence any of the effects of DEP or UD, therefore, the data are not shown.

### Influence on cell viability

**Figure 5:**
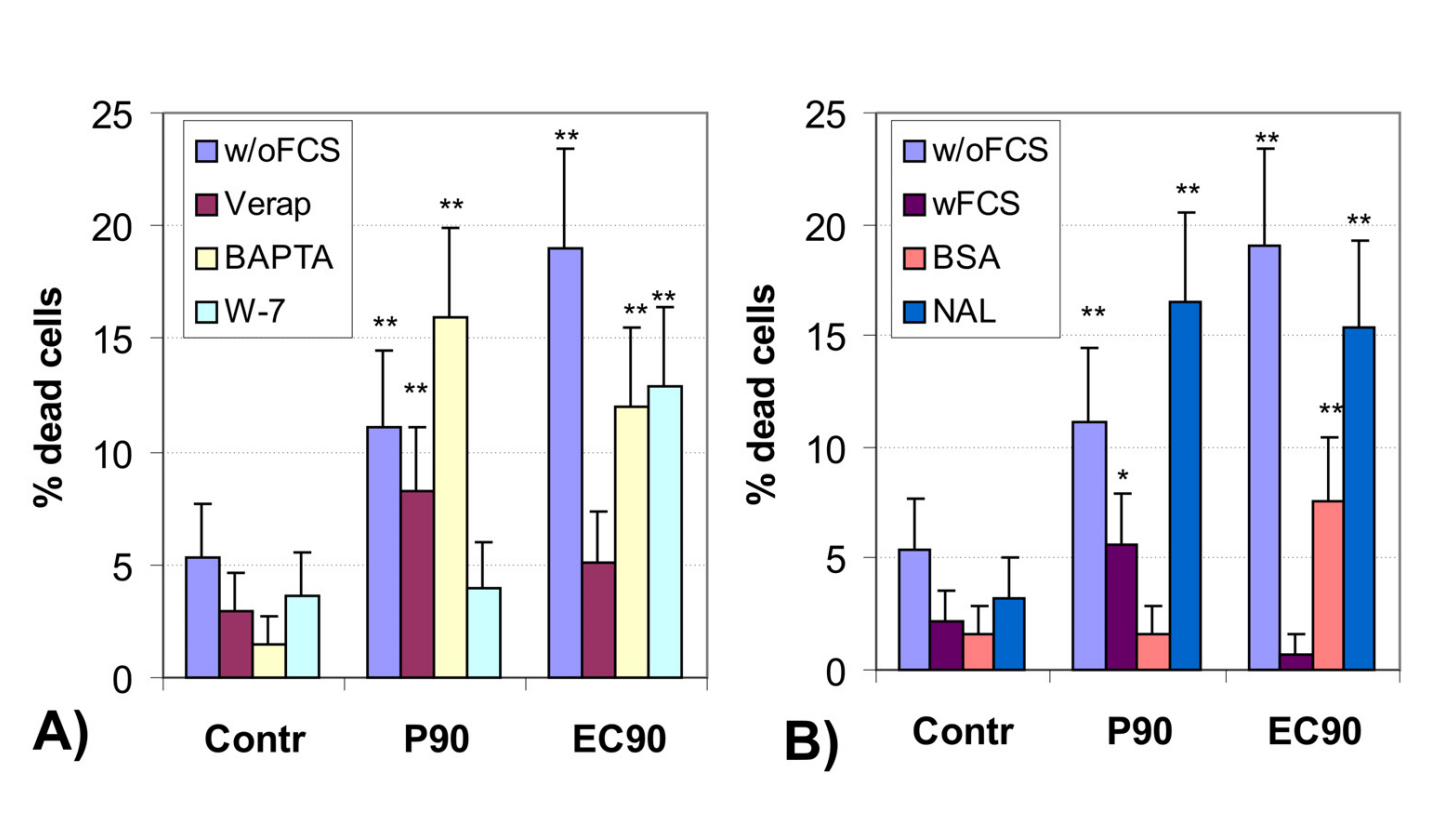
Cell viability (PI exclusion) of J774A.1 macrophages after 4 hours of incubation without (control) and with 320 *μ*g UFP/ml/million under different incubation conditions. A): without serum (w/oFCS), 100 *μ*M Verapamil, 20 *μ*M BAPTA-AM and 25 *μ*M W-7. B): w/oFCS, with 5% serum (wFCS), 1% bovine serum albumin (BSA) or 200 *μ*M Nacystelin (NAL); mean +/- SD, **: p < 0.01, *: p < 0.05.

After 4 hours incubation time none of the particles or drugs decreased cell viability to below 90% at a particle concentration of 100 *μ*g/ml/million cells. Figure 5 shows the results of the cytotoxicity test (PI exclusion) of the J774A.1 cells for a particle concentration of 320 *μ*g/ml and 4 hours incubation time. P90 and EC90 significantly enhanced the fraction of dead cells in medium w/o serum, which in part could be suppressed by Verapamil and W-7, but not by BAPTA-AM. In addition FCS and BSA, but not NAL reduced the fraction of dead cells after co-culture with P90 or EC90. Compared to control, there was no significant increase in the fraction of dead cells after co-culture with DEP or DU. An incubation time of 24 hours further enhanced the cytotoxic effect of the UFP (data not shown). The viability of the cells under control conditions (w/o particles) was about 90% with FCS, BSA or NAL and decreases to about 80% w/o FCS (p < 0.01 compared to 4 hours incubation time). 24 h incubation with the Ca-modulating drugs showed a reduced viability of 80% for Verapamil and BAPTA-AM, and 70% for W-7. This reflects non-physiological conditions of the Ca-modulating antagonists after long-term exposure. 320 *μ*g/ml P90 and EC90 raised the fraction of dead cells to up to 80% and the effect of the Ca-modulating drugs was not uniform. Long-term co-culture with DEP did not raise the fraction of dead cells, compared to control incubation conditions. UD caused a doubling of the fraction of dead cells, which could be inhibited in part by co-culture with Verapamil, W-7, and with NAL, but not with BAPTA-AM.

## Discussion and conclusion

### Necrosis caused by UFP

Figure 5 shows both the cytotoxic potential of the particles in inducing cell necrosis used in the study, together with the Ca-modulating antagonists, particularly after 4 hours incubation time. The ultrafine carbon black particles (P90 and EC90) had the most significant effect on cell viability, with a doubling of the fraction of dead cells after 4 hours and a triplication after 24 hours. EC90 particles caused more dead cells due to the higher specific surface area compared to P90. FCS and BSA both reduce the cytotoxic potential of P90 and EC90. In the presence of FCS or BSA, the particle surface may become coated with proteins, which may shield reactive sites on the particle surface and reduced the potential of causative oxidative reactions. Although serum free medium allows clearer experimental conditions in case of receptor dependent responses, the serum co-culture may be closer to physiological conditions in the lung, where surfactant coats and opsonizes the particles. On the other hand the inhibitory effects of the Ca-antagonists would not have been seen with the presence of serum in the medium. Long-term incubation of J774A.1 macrophages without serum induced a decrease in cell viability, showing that this by itself is a non-physiological condition. Interestingly, cell viability could be enhanced by the presence of serum or BSA in the medium. But for short time periods (4 hours), the absence of serum did not injure the cells.

DEP and UD produce less cytotoxicity compared to the pure carbon black particles, despite other reactive material may be present, like transition metals, and adsorbed organic substances. One reason for the lower cytotoxicity of DEP and UD may be the lower specific surface area. In a previous study we could show cytoskeletal dysfunctions caused by DEP and UD after 24 hours incubation time [20]. In this study different incubation conditions were used, where particles were dispersed in medium containing serum. It was shown that the particles form larger aggregates without serum proteins in the medium, causing particle clusters in the *μ*m-size range [54]. Such clusters are subject to different mechanisms of uptake and processing by the cells (phagocytosis versus endocytosis for smaller particles), although both are actin based mechanism. Phagocytosed materials are processed via the lysosomal digestion route, which is not the case after endocytosis. In addition the specific surface area is smaller for aggregated particles. It was also shown that organics adsorbed to the particle surface can lower the specific surface area, can shield reactive sites; therefore make the particles less toxic than plain particles [55]. We suggest here that the organic substances are stable when bound to the surface, in conjunction with low concentrations in the medium. The bound organics on the particles may cause a surface coating, which can shield reactive sites of the carbonaceous core.

### Influence of UFP on phagosome motion and on magnetic particle twisting

MTC studies investigate the transport of micron-sized phagosomes from two different perspectives. Relaxation directly monitors the coupling dynamics between phagosomes and the cytoskeleton [39] while phagosome twisting examines the mechanical integrity (viscoelastic properties, stiffness) of the cytoskeletal filaments, which are linked to the phagosomes [56]. Phagosome transport requires an intact cytoskeleton, intact phagosomes including motor proteins and energy (ATP) [33,57]. Additionally, intracellular calcium plays an important role for cytoskeletal functions and intracellular signaling in relation to immunological and non-immunological defense reactions [58,59]. Cytotoxic reactions can involve the energy metabolism of the cell, intracellular calcium transients [13], the dynamics of cytoskeletal filaments and the transport mechanisms of phagosomes [41,42]. A previous study has shown that destroying microfilaments by cytochalasin D resulted in a retarded relaxation and an increased stiffness [37,53]. Colchicine, which disrupts microtubuli, results in an accelerated relaxation and in a moderately increased stiffness. Figure 2A illustrates the retardation of relaxation caused by 100 *μ*g/ml P90, and the modulation by Verapamil and BAPTA-AM together with the retardation produced by 4 *μ*M Cytochalasin D. In comparison to the effects induced by the cytoskeletal drugs the dysfunctions produced by the carbonaceous UFP suggests destructions of microfilamentous structures and hence dysfunctions of the cytoskeleton. This is also in agreement with other studies which showed impairment of the phagocytic capacity (an actin based mechanism) after incubation with different types of UFP [19,20].

Intracellular calcium plays a central role in the modulation of the defense mechanisms causing inflammation. Further studies have shown that ufCB causes a transient increase of intracellular calcium, which can be inhibited by the calcium channel blocker Verapamil [13,60]. This was reflected in this study by the inhibition of ufCB induced cytoskeletal dysfunctions in the presence of Verapamil. Interestingly, the intracellular calcium chelator BAPTA-AM did not induce the expected suppression of cytoskeletal dysfunction. The reason for this lack of effect is not clear but suggests that not only the level of intracellular calcium is responsible for the ufCB induced dysfunctions.

Calmodulin (CaM) is a ubiquitous calcium binding protein that can bind to and regulate a multitude of different protein targets by affecting many different cellular functions. CaM mediates processes such as inflammation, metabolism, apoptosis muscle contraction and cellular movement. It has been shown that calmodulin has a direct impact on actin polymerization [61-63], acto-myosin binding and cross linking [64,65]. CaM is thought to activate the myosin light chain kinase (MLCK) and CaM kinase II by displacement of their auto inhibitory domains. Many of the proteins that bind CaM are unable to bind calcium themselves and as such use CaM as a calcium sensor and signal transducer. The fact that the CaM-inhibitor W-7 can suppress most of the cytotoxic reactions caused by ufCB co-culture shows that the calcium dependent signaling pathway is crucial for the cytotoxic effect and cytoskeletal dysfunctions. The lack of suppression after co-culture with EC90 particles may reflect their higher specific surface area.

The antioxidant Nacystelin did not inhibit the UFP induced dysfunctions of the cytoskeleton. Oxygen radicals cause an oxidation and depletion of cytoskeletal proteins (thiols), disruption of actin filaments and the inhibition of F-actin formation, and an actin cross linking [18]. The actin system is the most sensitive constituent of the cytoskeleton to the oxidant attack. Nacystelin can inhibit the thiol oxidation [66], but did not result in a suppression of UFP induced cytoskeletal dysfunctions. This shows that the thiol oxidation may not be the only response to UFP exposure (besides the modulation of the calcium metabolism), but the cross linking of actin filaments primarily may describe the retarded relaxation and the increased stiffness of the cytoskeleton.

The specific surface area of the particles is discussed to be a significant parameter relating to cytotoxic responses of ultrafine particles [16,67]. Our studies in part support this hypothesis. Cell viability seems to correlate with the specific surface area of the particles, where EC90 induces more dead cells compared to P90 or to UD and DEP. Impairment of relaxation and stiffening of the cytoskeleton seem not to depend on the specific surface area, but W-7 could inhibit the particle induced impairments in P90, but not in EC90, which has the higher specific surface area. Besides the different surface characteristics of DEP and UD compared to EC90 and P90, the lack of cytoskeletal dysfunctions of these materials seems to be in part induced by the smaller specific surface area.

## List of abbreviations

UFP ultrafine particles

ufCB ultrafine carbon black

PM2.5 particle mass with aerodynamic diameter <= 2.5 *μ*m

EC90 elemental carbon particles, 90 nm mobility diameter

P90 commercial carbon particles, Printex 90 (Degussa)

DEP diesel exhaust particles

UD urban dust

W-7 (N-(6-aminohexyl)-5-chloro-1-naphthalene-sulfonamide hydrochloride, 25 *μ*M, calmodulin inhibitor

CaM calmodulin

BAPTA-AM Ca2+ chelator

Verap Verapamil

BSA bovine serum albumin

FCS fetal calf serum

ATP adenosine triphosphate

PI probidium iodide

ROI region of interest

MTC magnetic twisting cytometry

RMF remanent magnetic field of cell/magnetic particle probe

b1 = B(1 min)B_0 _RMF after 1 min relaxation

b5 = B(5 min)B_0 _RMF after 1 min relaxation

## Competing interests

The author(s) declare that they have no competing interests.

## Authors' contributions

WM has long experience in MTC measurements and investigation of cytoskeletal functions using MTC. He has conducted the studies together with Dr. David Brown during a visit at the lab in Gauting/Germany. DB and VS have long experience in particle toxicology and the induction of reactive oxygen species. They have a particular knowledge and experience in the role of calcium transients in intracellular signalling pathways. They designed the studies and interpreted the results, and DB conducted the studies during a visit at the lab in Gauting/Germany. WK is a specialist in studies of ultrafine particle clearance and translocation, and in ultrafine particle toxicology. He produced the EC90 particles, characterized them and contributed to the study design and interpretation.
